# Comprehensive Cloning of *Prunus mume* Dormancy Associated MADS-Box Genes and Their Response in Flower Bud Development and Dormancy

**DOI:** 10.3389/fpls.2018.00017

**Published:** 2018-02-01

**Authors:** Kai Zhao, Yuzhen Zhou, Sagheer Ahmad, Zongda Xu, Yushu Li, Weiru Yang, Tangren Cheng, Jia Wang, Qixiang Zhang

**Affiliations:** ^1^Beijing Key Laboratory of Ornamental Plants Germplasm Innovation & Molecular Breeding, National Engineering Research Center for Floriculture, Beijing Laboratory of Urban and Rural Ecological Environment, Key Laboratory of Genetics and Breeding in Forest Trees and Ornamental Plants of Ministry of Education, School of Landscape Architecture, Beijing Forestry University, Beijing, China; ^2^Beijing Advanced Innovation Center for Tree Breeding by Molecular Design, Beijing Forestry University, Beijing, China

**Keywords:** *Prunus mume*, *DAM* genes, flower bud development, expression analysis, subcellular localization assessment, yeast two-hybrid, BiFC

## Abstract

*Dormancy Associated MADS-box* genes are SVP/MADs-box members and supposed to play crucial roles in plant dormancy of perennial species. In *Prunus mume*, *PmDAM6* has been previously identified to induce plant dormancy. In the current study, six *PmDAMs* were cloned in *P. mume* and functionally analyzed in yeast and tobacco to detect the roles of the genes paralogous to *PmDAM6*. The expression patterns together with sequence similarities indicate that *PmDAMs* are divided into two sub-clades within SVP group. Moreover, *PmDAMs* are verified to take part in the development of different plant organs, specifically the flower buds, in some intricate patterns. Furthermore, the PmDAM proteins are found to have special functions by forming corresponding protein complex during the development of flower bud and induction of dormancy. In particular, when PmDAM1 dominating in flower bud in the warm months, the protein complexes are consisted of PmDAM1 itself or with PmDAM2. With the decrease temperatures in the following months, PmDAM6 was found to be highly expressed and gradually changed the complex structure to PmDAM6-protein complex due to strong binding tendencies with PmDAM1 and PmDAM3. Finally, the homodimers of PmDAM6 prevailed to induce the dormancy. The results obtained in the current study highlight the functions of *PmDAMs* in the tissue development and dormancy, which provide available suggestions for further explorations of protein-complex functions in association with bud growth and dormancy.

## Introduction

For woody plants, bud formation is often concomitant with its ability to enter dormant state (Rohde and Bhalerao, 2007) and, therefore, flower bud development possesses a key status in flowering time alterations. To explain this important phenomenon, several models of floral organogenesis have been proposed for specimen plants (Theissen, 2001; Causier et al., 2010). *Prunus mume* has long been cultivated in China due to its significant ornamental and economic value. In northern China, it can bloom early in the spring after getting a quick release from dormancy. The roles of some gene families, in floral development, have been well characterized in *P. mume*, such as MADS-box, SBP-box, and TCP gene families (Xu et al., 2014, 2015; Zhou et al., 2016). Despite the fact that *DAM* genes, members of MADS-box gene family, are initially associated with plant dormancy and its release, functional associations of these genes remain poorly defined.

*DAM* genes were initially characterized in an *Ever-growing* (EVG) mutant of *Prunus persica*. This mutant has a deletion in EVG affecting up to four genes, thereby preventing terminal buds from endodormancy (Bielenberg et al., 2004). The map-based cloning analyses of EVG locus reveal that it includes six tandem duplication genes named Dormancy Associated MADS-box genes (*DAM1*–*6*) (Bielenberg et al., 2004, 2008). In perennials, such as *Prunus persica*, *Populus trichocarpa*, *Solanum tuberosum*, *Euphorbia esula*, and *Vitis vinifera*, *DAM* genes are all clustered to the SVP clade (Leseberg et al., 2006; Ruttink et al., 2007; Campbell et al., 2008; Horvath et al., 2008; Díazriquelme et al., 2009). For annuals, there are two members of SVP-type genes in *Arabidopsis thaliana* and *Lycopersicon esculentum*, while in *Oryza sativa* there are three (Alvarez-Buylla et al., 2000; Hileman et al., 2006; Arora et al., 2007). Some MIKC^C^ type genes are floral regulators such as *SVP* and *AGL24* of *A. thaliana* (Alvarez-Buylla et al., 2000). For various plant species, *SVP*-like genes were involved in the regulation of floral organs (Jaudal et al., 2013). In tobacco (*Nicotiana tabacum*), abnormal floral organs were caused by the ectopic expression of an *SVP*-like gene of soybean (*Glycine max*) (Zhang et al., 2016). Therefore, *SVP*-like genes might have significant roles in floral development. In *P. mume*, overexpression of *PmSVP1* and *PmSVP2* in *A. thaliana* results in a number of floral variations, including alteration in floral organ number, leaf-like sepals, large carpel out of perianth, additional trichomes and increased rosette branches (Li et al., 2017). There are eight *SVP* genes in *P. mume*, including six *PmDAMs*, which might inherit partial SVP functions in floral bud development.

The expression analyses of bud dormancy in some species, such as raspberry (*Rubus idaeus*), leafy spurge (*Euphorbia esula*), apple (*Malus domestica*), demonstrate that the transcripts of *DAM* genes exhibit differential accumulation during bud dormancy, which present transition-specifically up-regulated in endo-dormancy and eco-dormancy and down-regulated prior to its release (Mazzitelli et al., 2007; Campbell et al., 2008; Horvath et al., 2008; Kumar et al., 2016). In *Pyrus pyrifolia*, two *DAM* genes, *PpMADS13–1* and *PpMADS13–2*, show differential expressions during seasonal endo-dormancy induction (Ubi et al., 2010; Niu et al., 2015; Saito et al., 2015). The *DAM4*, *DAM5*, and *DAM6* of *P. persica* are identified during bud dormancy release by suppression subtractive hybridization, which are regulated, in part, by cold exposure (Leida et al., 2010). However, *DAMs* in peach are associated not only with seasonal elongation cessation but also bud formation (Li et al., 2009). Furthermore, a recent report reveals the involvement of *DAM* genes in reproductive processes and meristematic activities, thereby showing a putative role in regulating dormancy and flowering time in apple (Kumar et al., 2016). Six *DAM* genes in *P. mume* show endo-dormancy associated changes (Yamane et al., 2008; Sasaki et al., 2011; Xu et al., 2014; Yamane and Tao, 2015; Kitamura et al., 2016). In the lateral vegetative buds, *PmDAMs* show seasonal expression changes and keep high expression levels from July to October (Sasaki et al., 2011). In addition, PmDAM6 has been shown to interact with PmSOC1, suggesting its participation in dormancy transition, flower bud development and flowering time regulation in *P. mume* (Kitamura et al., 2016). Studying specific protein-protein interactions have become a powerful approach to understand the details of gene functions. Previous studies have implicated that MADS-box genes participate in plant development with the formation of protein complexes (Honma and Goto, 2001). Therefore, comprehensive understanding of these interactions can provide a framework to perceive the functional modes and the potential regulatory participation in bud growth and dormancy.

In the present study, six *PmDAMs* were cloned and phylogenetic relationships were investigated among them. Subcellular localizations of these proteins were ascertained and the expression patterns were observed in seven organs, flower buds (throughout an annual growth cycle) and at different stages of floral organ development. Furthermore, yeast two-hybrid and bimolecular fluorescence complementation (BiFC) were performed to confirm the interaction forms. Based on the foundation of gene expression patterns, a model among PmDAMs was constructed. The results of this research will contribute to build a groundwork for future studies in understanding the control of bud development and dormancy in plants.

## Materials and Methods

### Plant Material

*Prunus mume* ‘Sanlun Yudie,’ a double petal cultivar, was selected as the plant material from Beijing Forestry University, Beijing, China (40° 00′ N, 116° 18′ E). Samples were collected from seven different organs to analyze the expression levels of *PmDAMs*, including: full blooming flowers (March 22nd 2015); leaves on May 10th 2015; fruits and seeds on June 10th 2015; stems, flower buds and leaf buds on October 10th 2015 (Supplementary Table 1). The flower buds (from July 2015 to February 2016; collections every 30 days) were sampled to analyze the expression patterns of *PmDAMs* during flower bud development and dormancy.

The flower bud samples with consistent appearance were collected every 5–7 days and half of each sample was preserved in FAA (50% Ethanol, 2% Methanal, and 5% Glacial acetic acid). To perform paraffin section, thirty flower buds per stage were prepared to determine the development stages of flower buds. When 90% of the buds exhibited the same differentiation morphology (for example, the ‘petal initiation’) while the other buds (less than 3 buds) were in the prior stage (‘sepal initiation’), the stage was defined as petal initiation.

### Cloning of *PmDAM* Genes

Six *PmDAMs* have been identified in previous studies (Sasaki et al., 2011; Zhang et al., 2012; Xu et al., 2014). Specific primers of these genes were designed according to the CDS sequences from *P. mume* genome^1^. Total RNA was extracted from flower buds. Full-length cDNA of six *PmDAMs* and the plasmids of pMD^TM^18-T-*PmDAMs* were extracted by previous methods (Zhou et al., 2017). The primers and annealing temperatures of PCRs are shown in Supplementary Table 2.

### Phylogenetic Analyses

Multiple sequence alignment of six PmDAM proteins and 21 DAM proteins of other plants (six in *P. persica*, four in *Prunus pseudocerasus*, six in *Pyrus pyrifolia*, three in *M. domestica*, and two in *Camellia sinensis*) were used by DNAMAN 7.0 software with default parameters. The GeneBank accession numbers of these genes are shown in Supplementary Data 2. MEGA7.1 program was used to generate a phylogenetic tree of these DAM proteins and the other 19 type II MADS-box proteins in *P. mume* (Supplementary Data 3) with Maximum-likelihood (ML) Method. The parameters were set to default and the bootstrap values were set to 1000.

### Yeast Two-Hybrid Assay

Full-length coding sequences of *PmDAMs* were amplified via the PCR method with specific primers (Supplementary Table 5). To clone these sequences into pGBKT7(bait) vectors and pGADT7 (prey) vectors (Clonetech, United States) at the *Eco*RI and *Bam*HI sites respectively, In-Fusion HD Cloning System (Clonetech, United States) was used. Yeast 2 hybrid assay was performed according to the previous method (Zhou et al., 2017). The screenings for each interaction were applied in triplicate.

### Subcellular Localization Assessment

The coding sequences of *PmDAMs* were cloned into pSuper1300-GFP plasmid using In-Fusion HD Cloning Kit System (Clonetech, United States) and obtained the 35S::PmDAM::GFP fusion vectors. Specific primers used for subcellular localization assessments are listed in Supplementary Table 4. Before transferred to *Nicotiana benthamiana*, the vectors were checked through sequencing. Agroinfiltration was carried out on the leaves of *N. benthamiana*. The plasmids were transformed into *A. tumefaciens* strains (GV3101) and cultured (at 28°C) in Luria-Bertani medium containing kanamycin (50 μg/ml), gentamicin (50 μg/ml) and rifampicin (50 μg/ml). After harvested, the bacteria were resuspended in an infiltration buffer (10 mM MES, 10 mM MgCL_2_, 150 μM acetosyringone) and the suspension concentration of bacteria was calculated through spectroscopy at 600 nm optical density and the final concentration was adjusted to 0.5–0.8. This mixture was kept for 2 h at room temperature in the darkness. Bacterial suspension was infiltrated into the leaves (through abaxial surfaces) using syringes and the leaves were taken after 2 days to ascertain subcellular localization. Leaf tissues of *N. benthamiana* were dyed with DAPI for the precise assessment of nucleus position. Finally, the leaves were examined under Leica TCS SP8 Confocal Laser Scanning Platform. Excitation/emission settings were 405nm for DAPI and 488 nm for GFP.

### BiFC Assay

*PmDAMs* were cloned into pCambia1300-YFP-N and pCambia1300-YFP-C vectors. Co-expression was executed on *N. benthamiana* leaves as described in subcellular localization assessments. Chimeric fluorescence from expressed fusion proteins was checked 2 days after infiltration. Images were generated through Leica TCS SP8 Confocal Laser Scanning Platform. YFPs were excited at 514 nm. Specific primers for BiFC analysis were used (Supplementary Table 6).

### Quantitative Real-Time PCR

To investigate the expression patterns of *PmDAMs* genes in different organs and flower bud development, real-time quantitative PCR experiments were performed via former method (Zhou et al., 2017). The primers of RT-qPCR are shown in Supplementary Table 3. For all quantitative real-time PCR reactions, three biological replicates were carried out and each replicate had three technical repeats. The reference gene for these reactions was *PmPP2A* (*protein phosphatase 2A*) (Wang et al., 2014). The correlations and significant tests of expression patterns were calculated by R, following the method of Spearman and ANOVA, respectively.

## Results

### Cloning of *DAM* Genes in *P. mume*

There were six *DAM* genes in the *P. mume* genome named *PmDAM1*, *PmDAM2*, *PmDAM3*, *PmDAM4*, *PmDAM5*, and *PmDAM6* (the accession numbers are shown in Supplementary Data 1). The CDS sequences of *PmDAM1-6* were of 708bp, 723bp, 708bp, 669bp, 705bp, and 726bp, encoding for 235, 240, 235, 222, 234, and241 amino acids, respectively. According to BLAST analysis, all *PmDAM*s exhibited high similarity and consistency to their orthologs. All these genes contained conserved MADS-box domain and K-box domain as confirmed by Interpro (Supplementary Figure 1).

### Multiple Sequence Alignment and Phylogenetic Analyses

**FIGURE 1:**
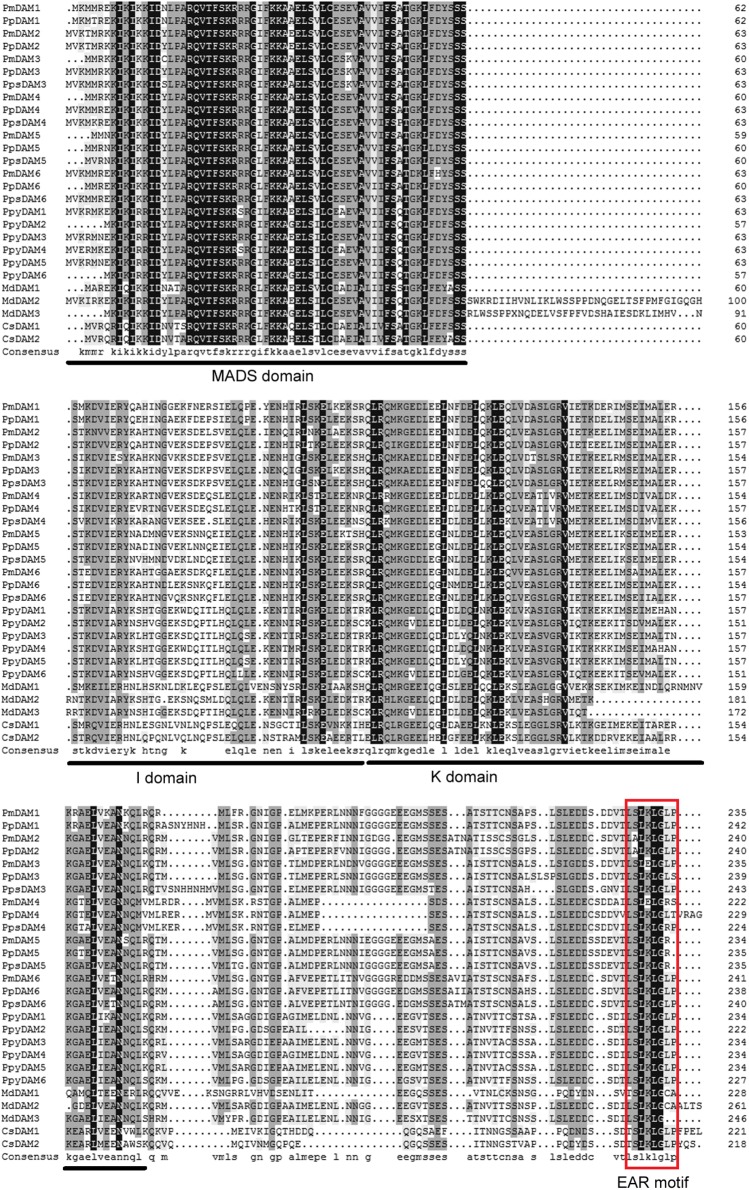
Multiple sequences alignment of DAM genes from *P. mume* and other species. The MADS domain, I domain, and K domain are shown by lines on bottom of the alignment. EAR (ethylene-responsive element-binding factor-associated amphiphilic repression) motif is shown by red rectangular. The GeneBank accession numbers of genes used in alignment are showed in Supplementary Data 2.

**FIGURE 2:**
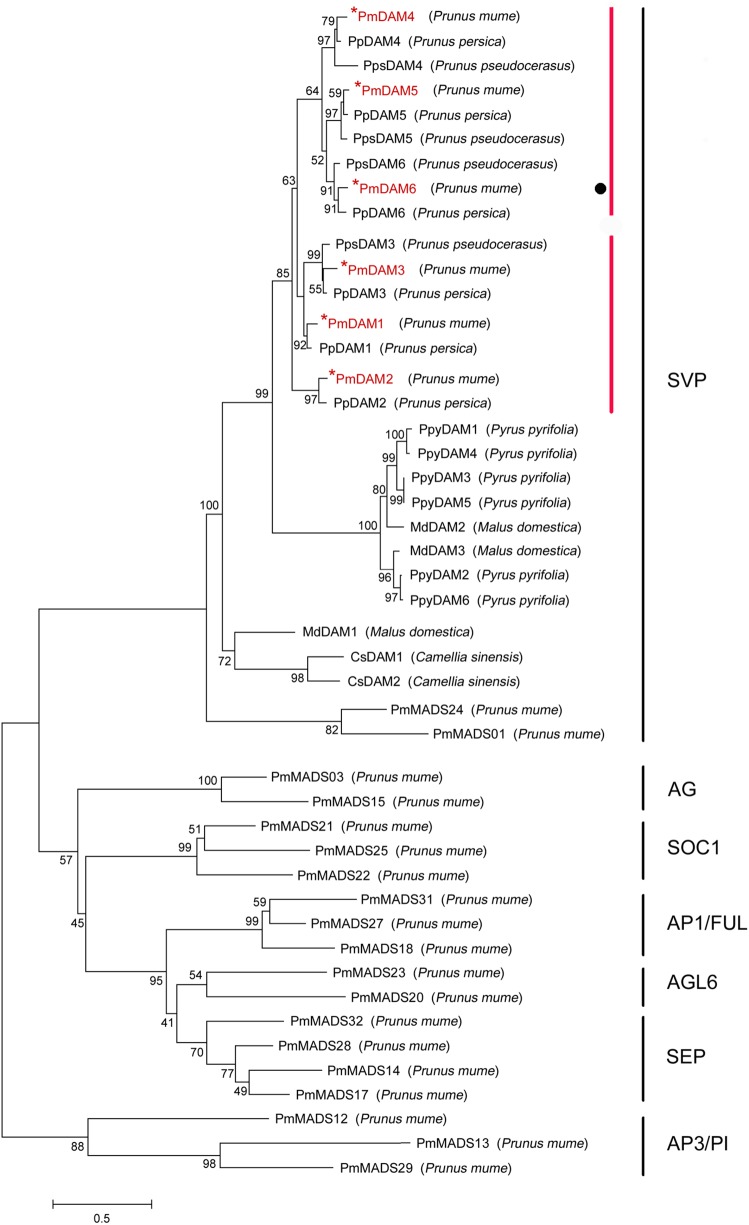
Phylogenetic tree of DAM proteins and other 19 type II MADS-box proteins in *P. mume*. The sequences of these proteins were shown in Supplementary Data 3. The types of *P. mume* MADS-box proteins in different clusters were named according the phylogenetic analysis of *P. mume* MADS-box gene family (Xu et al., 2014). Numbers above branches represented bootstrap value. DAMs in *P. mume* are colored by red.

Multiple sequence alignment of DAMs in different species was accomplished by DNAMAN 7.0 program. The consistency value of DAM genes among *P. mume* and other species was 60.94%. In PmDAMs, the MADS domain showed high conservation at the N-terminal of protein sequences, whereas the K domain showed moderate conservatism, and the putative I domain expressed little conservatism (**Figure 1**). At the C-terminal of these proteins, there was a conserved EAR motif. The phylogenetic tree of DAM genes exposed that six *P. mume* DAM genes belong to the SVP clade of MADS-box gene. These genes were first clustered with DAM genes from other *Prunus* species (*P. persica* and *P. pseudocerasus*), then with other Rosaceae plants (*Malus domestica* and *Pyrus pyrifolia*), and finally got close to other species (**Figure 2**). These six genes had high homology. *PmDAM1*, *PmDAM2*, and *PmDAM3* were clustered in one branch; *PmDAM4*, *PmDAM5*, and *PmDAM6* were gathered in the other branch.

### Expression Levels of *PmDAMs* in Different Organs

**FIGURE 3:**
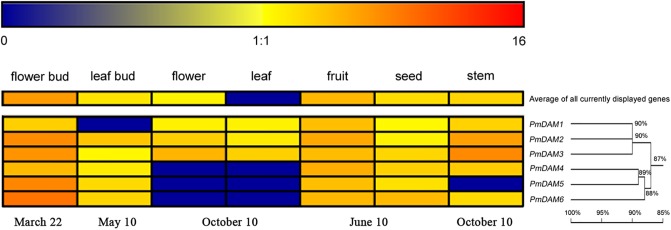
Expression profiles of *P. mume DAM* genes in seven organs. The expression levels of *PmDAMs* were gained using quantitative real-time PCR method. The log2-transformed counts of the expression levels of *PmDAMs* were represented by the *color scale. Red* indicated high expression levels and *Blue* suggested low levels. Homology tree was constructed by DNAMAN 7.0 program using six *P. mume DAM* genes. Numbers above branches represented bootstrap value.

In order to explore the roles of *PmDAMs* in different organs, the expression patterns of six *PmDAMs* in flower bud, leaf bud, flower, leaf, fruit, seed, and stem were studied using RT-qPCR. Six *PmDAM*s expressed in all organs. These genes were predominantly detected in flower buds and fruits, moderately expressed in leaf buds, flowers, fruits, seeds, and stems, while poorly expressed in leaves (**Figure 3**). Based on their expression patterns in seven organs, the *PmDAM*s were divided into two groups which were similar with the results of the homology tree. The transcripts of *PmDAM1*, *PmDAM2*, and *PmDAM3* were highly expressed in flower bud, fruit, and stem, and were mildly detected in leaf bud, leaf, flower, fruit, and seed. For *PmDAM4*, *PmDAM5*, and *PmDAM6*, the expression levels of these three genes were notable in flower bud, mild in leaf bud, fruit, seed, stem, and faint in flower, leaf.

### Expression Analyses of *PmDAMs* during Flower Bud Development

**FIGURE 4:**
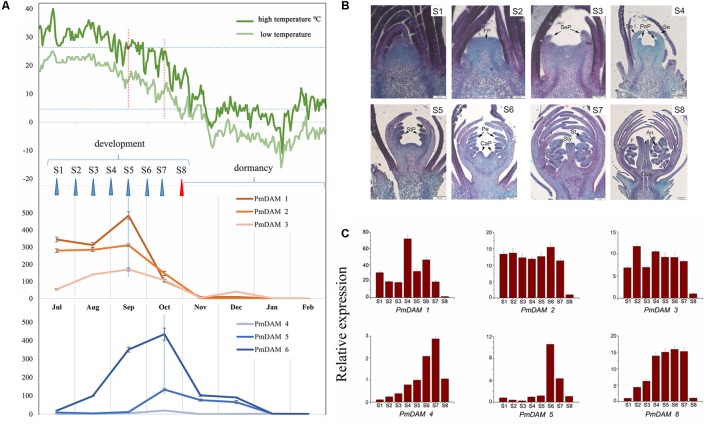
Expression patterns of *PmDAMs* during *P. mume* flower bud growth. **(A)** Expression patterns of *PmDAMs* in flower buds development with the changes of temperatures. The green lines indicated the corresponding temperatures, the deep on showed the highest temperature and the light green show the lowest ones. **(B)** Flower bud statuses of *P. mume*. The flower bud development was divided into eight stages (S1–S8): undifferentiation (S1), flower primordium formation (S2), sepal initiation (S3), petal initiation (S4), stamen initiation (S5), pistil initiation (S6), ovule development (S7), anther development (S8). FP, Flower primordium; SeP, Sepal primordium; Se, Sepal; PeP, Petal primordium; Pe, Petal; StP, Stamen primordium; St, Stamen; CaP, Carpel primordium; Ca, Carpel; Sty, Style; An, Anther; F, Filament; Ova, Ovary; Ovu, Ovule; Po, Pollen. **(C)** Expression patterns of *PmDAMs* during *P. mume* floral bud development.

In Beijing, North of China, the flower bud development of *P. mume* ‘Sanlun Yudie’ occurs from June to July; begins to differentiate from July to November; enters dormancy from September to October; retains dormant from November to January, and breaks dormancy in February. The expression of *PmDAM*s showed a certain pattern with the changes of temperatures (**Figure 4A**), and exhibited peak expressions in different months with two grouping trends. *PmDAM1*, *PmDAM2*, and *PmDAM3* formed one group, showing high expression levels from July to October, and their expression levels were fairly low from November to February. *PmDAM4*, *PmDAM5*, and *PmDAM6* formed the other group, exhibiting the highest transcript levels in October (**Figure 4A**). The latter group exhibited an increasing expression trend from July to October, which then gradually declined from November to February. Expressions of *PmDAM4*, *PmDAM5*, and *PmDAM6* were up-regulated during dormancy induction and down-regulated during bud dormancy breakup. Indeed, the correlation between *PmDAM1-3* reached a value above 0.83, especially *PmDAM1* and *PmDAM2* got a value of 0.97 with a significant positive correlation. In addition, *PmDAM4- PmDAM6* showed positive correlations with value up to 0.81.

According to the paraffin section analyses (**Figure 4B**), there were eight stages of flower bud development in *P. mume* (S1–S8). All six *PmDAMs* were expressed during the differentiation of flower buds (**Figure 4C**). *PmDAM1*, *PmDAM2*, and *PmDAM3*. were expressed in first seven stages. The transcript of *PmDAM1* was prominently detected in S4. *PmDAM2* and *PmDAM3* showed similar expression levels during S1–S7. *PmDAM4*, *PmDAM5* and *PmDAM6* exhibited stage-specific expression profiles. The expression levels of *PmDAM4* and *PmDAM5* continuously increased during S1–S6. *PmDAM6* was up-regulated in S1–S5. All *PmDAMs* were fairly expressed in S9.

### Subcellular Localization Assessment

**FIGURE 5:**
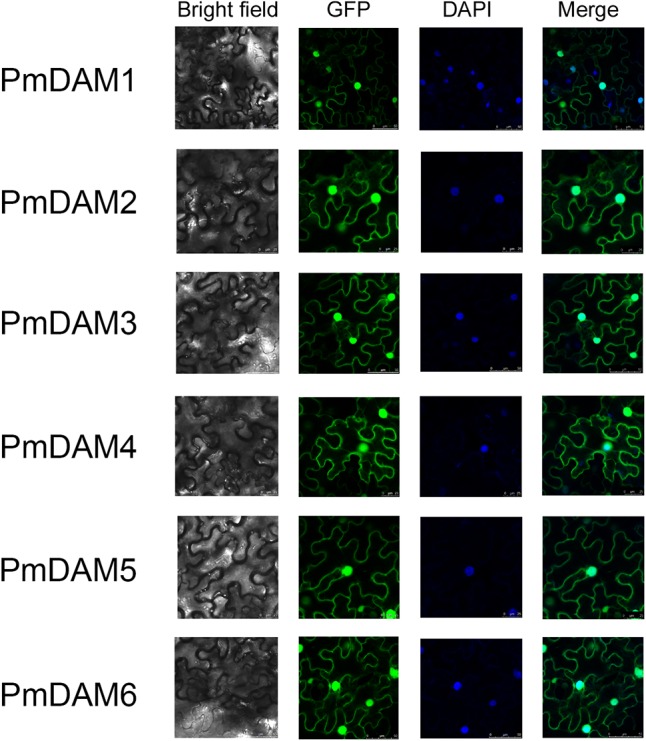
Subcellular localization of six PmDAMs. To determine the exact position of PmDAMs within the cell, subcellular localization experiments were performed using leaf tissues of *N. benthamiana*. The green fluorescent showed protein position. The blue fluorescent presented the nuclei position. The merge pictures of PmDAMs were formed by the pictures of GFP and DAPI.

To determine the exact positions of PmDAMs within the cell, subcellular localization experiments were performed. The vectors with GFP, under control of 35S promoter, were temporarily overexpressed in the leaves of *N. benthamiana*. Confocal imaging revealed the colocation of all the PmDAMs with the nucleus marker, 4′, 6-diamidino-2-phenylindole dihydrochloride (DAPI), in the parenchyma cells of abaxial epidermis of *N. benthamiana* leaves. Thus, subcellular location assay indicated that PmDAMs were mainly expressed in cell nucleus (**Figure 5**).

### Protein–Protein Interactions (Yeast Two-Hybrid Assay) among DAM Proteins in *P. mume*

**FIGURE 6:**
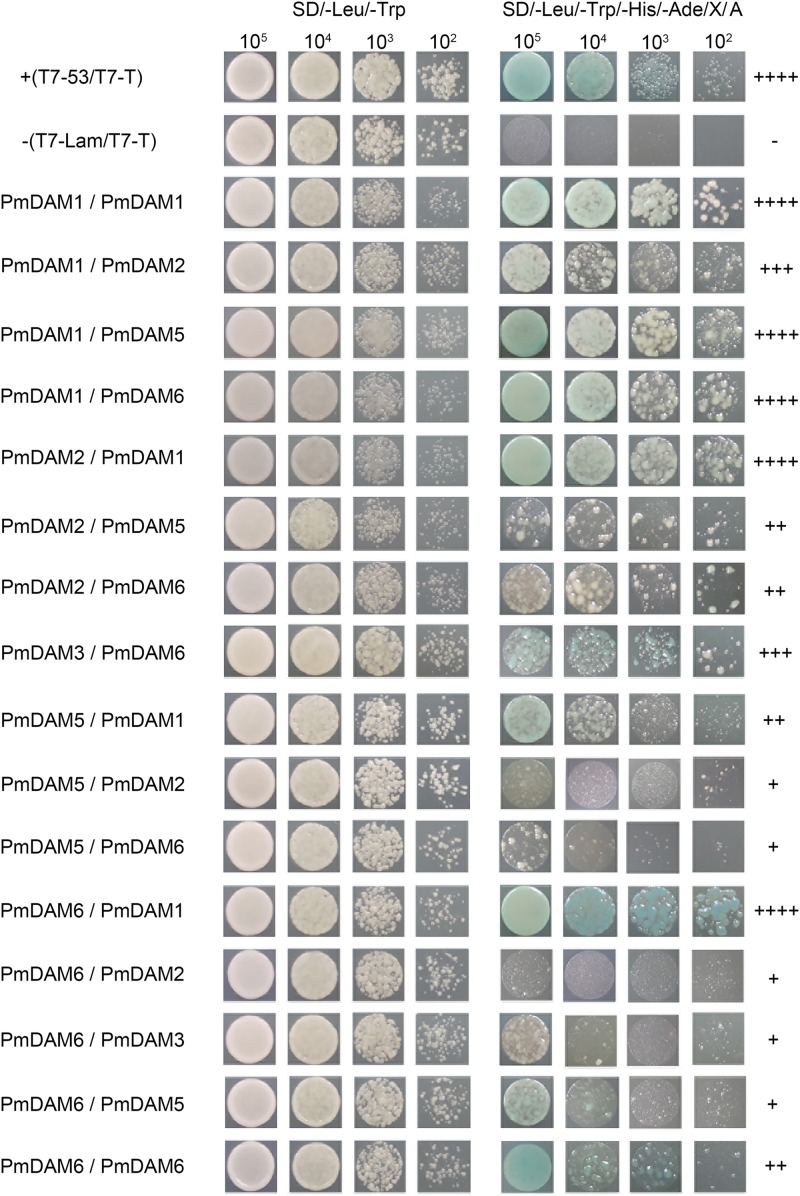
Protein–protein interactions (yeast two-hybrid) among PmDAMs. To determine the interaction model of PmDAMs, Yeast two-hybrid assays were performed. T7-53/T7-T was positive control, and T7-Lam/T7-T was negative control. The symbol (+) represents the capacity of the reaction. The more numbers of the symbol (+), stronger is the capacity of the reaction.

To investigate the interaction model of *P. mume* DAM genes, yeast two-hybrid assay was performed to clarify the protein complexes formed by specific PmDAMs. The six baits of *PmDAMs* did not show auto activation and toxicity. **Figure 6** shows the interaction patterns of PmDAMs. PmDAM1 and PmDAM6 could form a homologous dimer, but PmDAM2, PmDAM3, PmDAM4, and PmDAM5 could not. PmDAM1 showed strong interactions with PmDAM2, PmDAM5, and PmDAM6. PmDAM2 could strongly dimerize with PmDAM1, and the abilities to interact with PmDAM5 or PmDAM6 were moderate. In addition, PmDAM3 could interact only with PmDAM6. PmDAM4 neither formed homodimers nor showed interactions with other PmDAMs. PmDAM5 could form heterodimers with PmDAM1, PmDAM2, and PmDAM6. PmDAM6 showed a strong interaction with PmDAM1 and weak interactions with PmDAM2, PmDAM3, and PmDAM5.

Therefore, only PmDAM1 and PmDAM6 could form homodimers in PmDAMs. PmDAM1, PmDAM2, PmDAM5, and PmDAM6 could dimerize with others. PmDAM1 exhibited strong interactive capability to form homodimer as well as heterodimers. Moreover, PmDAM6 could form heterodimers with other PmDAMs except PmDAM4.

### BiFC Confirmations of Protein–Protein Interactions

**FIGURE 7:**
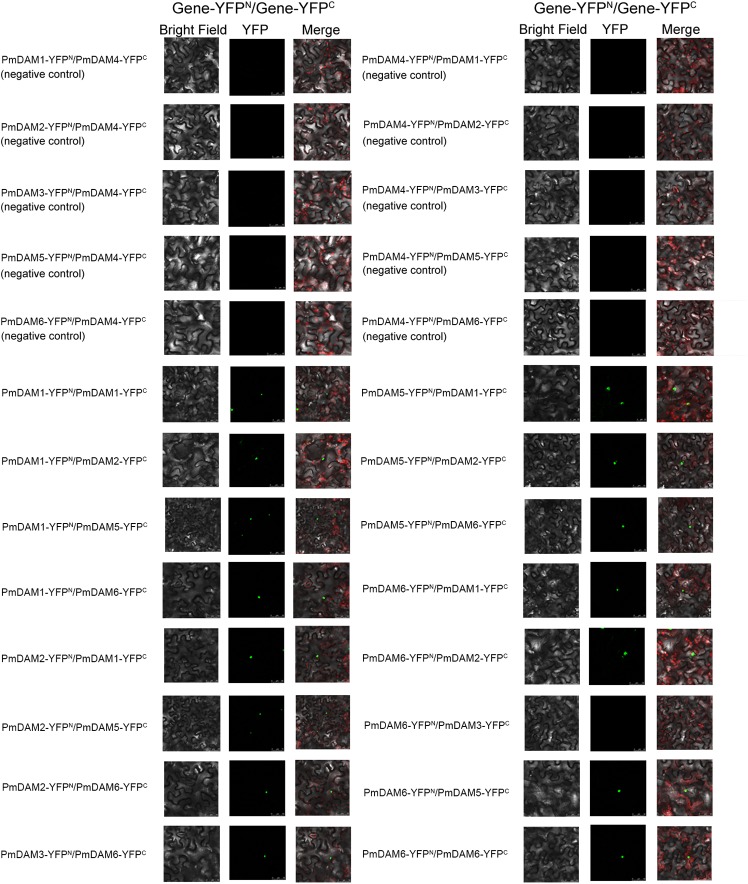
BiFC analysis of the protein interactions among PmDAMs which had been confirmed by yeast two-hybrid assays. In every interactions, the two proteins were fused with either the C or N terminus of yellow fluorescent protein (YFP; designated as YFP^C^ or YFP^N^, respectively). Different combinations of the fused constructs were co-transformed into leaf cell of *N. benthamiana*, and then the cells were observed by confocal microscopy as described in “Materials and Methods.” PmDAM4, a homolog with all PmDAMs, had no interaction with other PmDAMs and was chosen as negative controls. Bright field and YFP were excited at 514 nm. The green fluorescent presented protein position. The red fluorescent showed the chloroplast.

Protein–protein interactions between PmDAMs were further studied by BiFC assay with a yellow fluorescent protein (YFP). YFP fluorescence was localized to the nuclei. There was no interaction between YFP^N^/YFP^C^ and PmDAMs-YFP^C^/PmDAMs-YFP^N^ (Supplementary Figure 2). Thus, the interactions between PmDAM4 and six PmDAMs were used as the negative controls. In these control experiments, no YFP fluorescence was detected. In eight pair-wise reactions (16 reactions) among PmDAMs, there were 15 positive results in accordance with the yeast two-hybrid, indicating the interactions among PmDAMs (**Figure 7**). The only combination showing no fluorescent was PmDAM6-YFP^N^/ PmDAM3-YFP^C^.

## Discussion

### *PmDAM*s Were Splited under SVP Group during Evolution

Members of the SVP-like gene family have been identified in a wide range of species and have been shown to perform diverse functions. In Arabidopsis, the family is represented by two paralogous genes, SVP and AGL24, performing opposite functions during floral initiation. *DAM* genes are widely found in perennials, and evolved from *SVP* genes (Horvath et al., 2010; Sasaki et al., 2011). *DAM* genes were first identified in an ever-growing mutant of peach. As Mei being a relative specie to peach under rosaceae, PmDAMs show quite similar structures with PpDAMs. However, for the pear, the DAMs stand alone in the other branch. Therefore, peach and Mei both might undergo the same evolutionary process in rosaceae. This indicates the same ancestor but different appearance time for the *DAM* genes or there may exist functional diversification between species. These six *PmDAMs* may evolve from their own duplicative events within *Prunus*. In combination with the analysis of homology tree, six *PmDAMs* had been divided into two clusters. This classification may suggest new functional diversity during the evolutionary processes of these genes.

### Discovery of PmDAM Interologs from Paralogs

**FIGURE 8:**
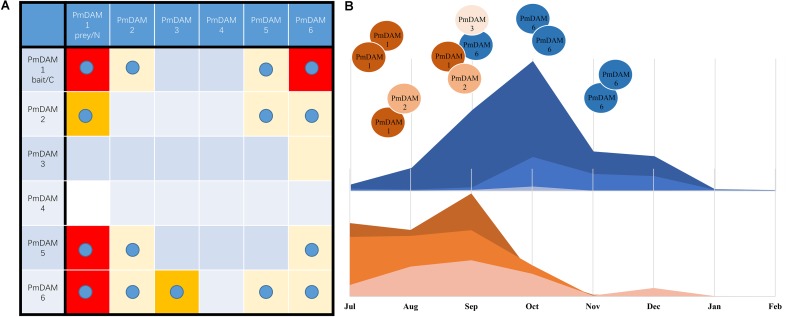
A hypothesis of molecular model of PmDAMs in flower bud growth and induction of dormancy. **(A)** Overview of interaction results for PmDAMs in yeast and tobacco leaves. The blue ball means the interaction was confirm by BiFC. And the color reflected the interactions in Y2H. Red blocks displayed the most furious binding intensities. **(B)** A summary of complex changing into the induction of dormancy.

Interactions among different or similar proteins are of great interest to know the ultimate details of the functioning, serving as building blocks to drive biological processes in the molecular networks. In Arabidopsis, the interaction matrix is presented with nearly all members in MADS-box transcription factor family (de Folter et al., 2005). However, DAMs are not involved in this model species. The multiple members in PmSVPs bring more functional diversities in some processes. For example, AtSVPs interact with SEP, SOC and FUL, the loss or increase of SVP members may lead to the aberrance of flower. Although PmDAM6 in Japanese Apricot (*P. mume*) showed auto-activation in the previous research and when a partial PmDAM6 protein was used as a bait, no interactions were observed among PmDAMs (Kitamura et al., 2016). However, PmDAMs of *P. mume* ‘Sanlun Yudie’ showed no autoactivation and were used as baits in yeast two-hybrid assays. In this study, complementary assay clearly demonstrates the interaction among six PmDAMs. Based on yeast two-hybrid assay, PmDAM proteins except PmDAM4 formed dimers with one or more PmDAMs, and there presented different intensity levels between corresponding protein molecules (**Figure 8A**). Considering the fact that PPI reflects the interactions of molecules, the protein, with strong binding capacity, would supersede the weak one. This may be caused by the variants from the C terminal of proteins (**Figure 1**).

Previous studies have proved the associations of *DAM* genes with bud dormancy. *PpDAM*s are found in leaf, root, stem, bud, and fresh fruit. These genes except *DAM6* are also detected in flower, and these genes except *DAM1* are expressed in seed (Li et al., 2009). In *P. mume*, six *DAM* genes were all expressed in different plant parts (i.e., flower bud, leaf bud, flower, leaf, fruit, seed, and stem). The difference of expression profiles between these two *Prunus* species might be due to the diversity in the state of flower and seed. In *P. mume*, *PmDAM1*, *PmDAM2*, and *PmDAM3* might function in these seven organs, especially the flower bud, fruit, and stem due to the formation of PmDAM1 homo- or heterodimers by PmDAM1 and PmDAM2. *PmDAM4*, *PmDAM5*, and *PmDAM6* might play significant roles in flower bud, leaf bud, fruit, seed, and stem, particularly in the flower bud. Combined with our results, PmDAMs could form different complexes in different organs.

### Protein Complexes of PmDAMs Alternating in Flower Bud Growth and Dormancy

The change of plant condition from development to dormancy involves quite a number of genes (Howe et al., 2015; Hao et al., 2017), thus, making it more difficult to know the internal changes in the dormant tissues. In peach, six *DAM* genes play roles in seasonal dormancy of buds (Bielenberg et al., 2008). These genes show different expression patterns in terminal tissues of field-grown peach trees throughout an annual growth cycle (Li et al., 2009). *PpDAM1*, *2*, and *4* are associated with bud formation and seasonal elongation cessation and the expression levels of *PpDAM5* and *PpDAM6* gradually increase during the autumn and exhibit peak levels in winter (Li et al., 2009). As shown in the **Figure 8B**, *Prunus mume* underwent similar changes. In the warm months, PmDAM1 and PmDAM2 dominated in the flower buds. The protein complex of PmDAMs consisted of PmDAM1 or PmDAM2, which own a similar structure with PmSVP (PmMADS1 and PmMADS24), suggesting functional similarity to SVPs (Wu et al., 2017). But with the expression of PmDAM6, which have a stronger binding ability with PmDAM1, the inner complex turned more complicated. Nonetheless, the flower buds continued developing under a relatively high level of PmDAM6 (**Figure 4**, S5–S7). Thus, a hypothesis was proposed that the two groups of PmDAMs possess opposite functions, the complicated protein complexes restrict the performance of protein functions. At last, until the PmDAM6 was in the lead, flower buds began to fall into dormancy with a homodimer of PmDAM6. On the whole, PmDAM protein complexes experience a switch in the release of dormancy by PmDAM6.

## Conclusion

In this study, six cloned *PmDAM*s were clustered into two functional groups (*PmDAM1-3 and PmDAM4-6*, respectively). As indicated by the experimental results, the transcripts of *PmDAM1* begin to accumulate in the warm season, while *PmDAM6* increases gradually with the drop of temperature to induce bud dormancy. Moreover, *PmDAM6* is found to act oppositely against *PmDAM1-3*. It is verified that the core abilities of protein–protein interactions existed among PmDAM1, PmDAM6 and PmDAM5 proteins. Therefore, it can be concluded that PmDAMs, which are located in the nucleus, impact synthetically the growth and dormancy of the flower bud. During this process, the protein complex formed mainly by PmDAM1 and PmDAM2 changes into a more complicated structure consisted of PmDAM1, PmDAM2, PmDAM3 and PmDAM6, which then evolves to PmDAM6 protein dominating complex.

The present study provides available information for further investigations on the functions of *DAM* genes in flower bud growth and dormancy. Based on the findings of the current study, further efforts can be contributed to figure out the interactions of *DAM* with other functional MADS-box members in the next future.

## Author Contributions

YZ and QZ designed the experiments. YZ completed the experiments. KZ wrote the manuscript. ZX contributed to identify the stages of flower bud differentiation and provided the sequences of 19 *P. mume* MADS-box protein. SA improved the manuscript. KZ, WY, TC, and JW analyzed the data. All authors read and approved the final manuscript.

## Conflict of Interest Statement

The authors declare that the research was conducted in the absence of any commercial or financial relationships that could be construed as a potential conflict of interest.

## Footnotes

1 http://prunusmumegenome.bjfu.edu.cn/
