# Supplementary material for: Comprehensive Cloning of Prunus mume Dormancy Associated MADS-Box Genes and Their Response in Flower Bud Development and Dormancy
Source: Front Plant Sci. 2018 Feb 1;9:17. doi: 10.3389/fpls.2018.00017 (PMC5800298; doi:10.3389/fpls.2018.00017)
Supplement: Supplementary file 1 [file Data_Sheet_1.docx]

***Supplementary Material***

**Comprehensive Cloning of *Prunus mume* Dormancy Associated MADS-box genes and Their Response in Flower Bud Development and Dormancy**

Kai Zhao^1†^, Yuzhen Zhou^1†^, Sagheer Ahmad^1^, Zongda Xu^1^, Yushu Li^1^, Weiru Yang^1^,Tangren Cheng^1^, Jia Wang^1^ and Qixiang Zhang^1,2*^

1 Beijing Key Laboratory of Ornamental Plants Germplasm Innovation & Molecular Breeding, National Engineering Research Center for Floriculture, Beijing Laboratory of Urban and Rural Ecological Environment, Key Laboratory of Genetics and Breeding in Forest Trees and Ornamental Plants of Ministry of Education, School of Landscape Architecture, Beijing Forestry University, Beijing, China, 2 Beijing Advanced Innovation Center For Tree Breeding by Molecular Design, Beijing Forestry University, Beijing, China

*** Correspondence:** Qixiang Zhang, [zqxbjfu@126.com](mailto:zqxbjfu@126.com)

†These authors have contributed equally to this work

# Supplementary Figures and Tables

## Supplementary Figures

**Supplementary Figure 1.** **The CDS and deduced amino acid sequences of *PmDAMs*.** The red rectangular region indicates MADS domain, while the yellow rectangular region indicates I domain, and the blue rectangular region indicates K domain.


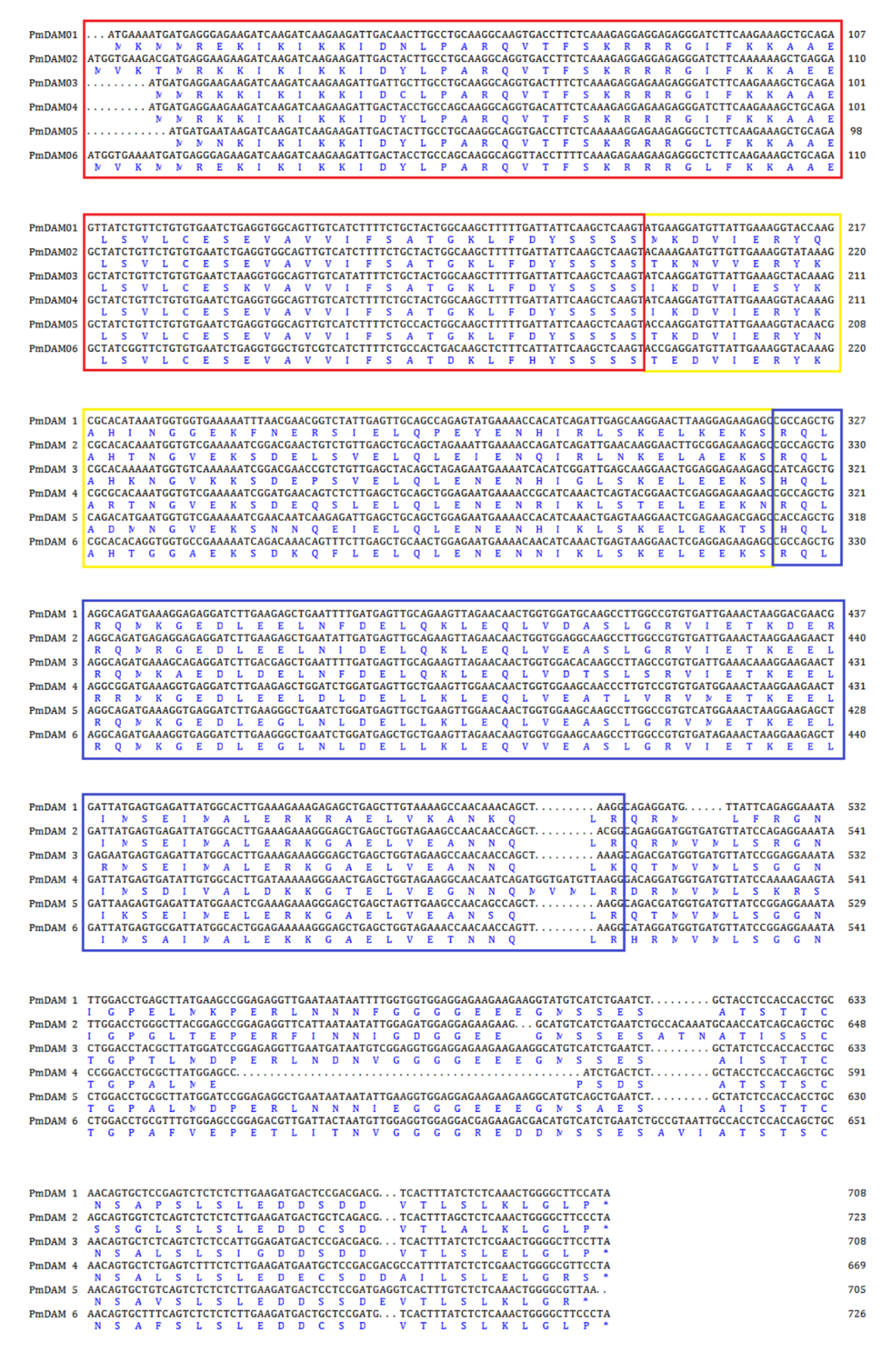


**Supplementary Figure 2. Control experiments of BiFC assays.** In every interactions, the two proteins were fused with either the C or N terminus of yellow fluorescent protein (YFP; designated as YFP^C^ or YFP^N^, respectively). Different combinations of the fused constructs were co-transformed into leaf cell of *N. benthamiana*, and then the cells were observed by confocal microscopy. There was no interaction between YFP^N^/YFP^C^ and PmDAMs-YFP^C^/PmDAMs-YFP^N^. Bright field and YFP were excited at 514 nm. The red fluorescent showed the chloroplast position.


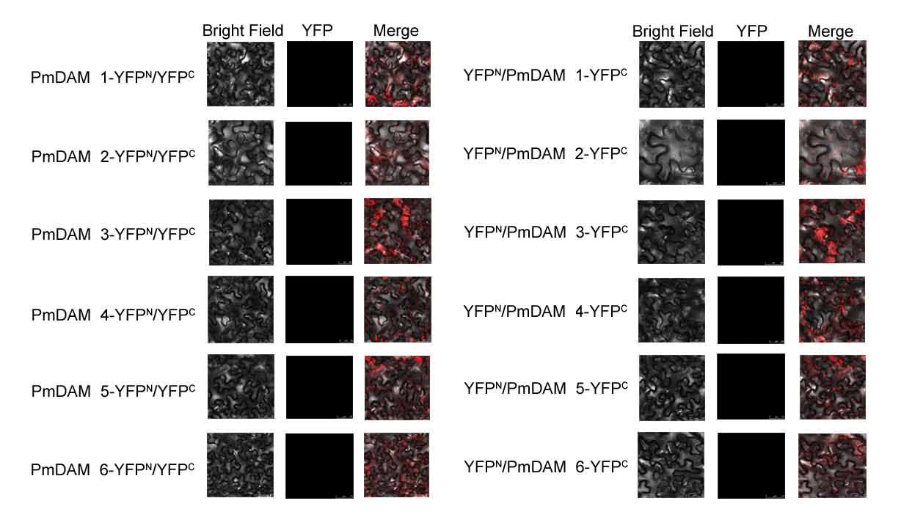


## Supplementary Tables

**Supplementary Table 1.** Plant material of seven different organs.

| **Organ** | **Time** |
| --- | --- |
| Flower (full blooming) | March 22 2015 |
| leaf | May 10 2015 |
| stem | October 10 2015 |
| flower bud | October 10 2015 |
| leaf bud | October 10 2015 |
| fruit | June 10 2015 |
| seed | June 10 2015 |

**Supplementary Table 2. Primers used for cloning.**

| Gene | Forward primer | Reverse primer | Annealing temperature |
| --- | --- | --- | --- |
| *PmDAM1* | 5'ATGAAAATGATGAGGGAGAAG3' | 5' TTATGGAAGCCCCAGTTTGAG3' | 52 ℃ |
| *PmDAM2* | 5'ATGGTGAAGACGATGAGGAAG3' | 5' TTAGGGAAGCCCCAGTTTGAG3' | 56 ℃ |
| *PmDAM3* | 5'ATGATGAGGAAGAAGATCAAG3' | 5' TTAAGGAAGCCCCAGTTCGAG3' | 51 ℃ |
| *PmDAM4* | 5'ATGGTGAAAATGATGAGGGAG3' | 5' TTAGGAACGCCCCAGTTTGAG3' | 54 ℃ |
| *PmDAM5* | 5'ATGATGAATAAGATCAAGATC3' | 5' TTAACGCCCCAGTTTGAGAGA3' | 47 ℃ |
| *PmDAM6* | 5'ATGGTGAAAATGATGAGGGAG3' | 5' CTAGGGAAGCCCCAGTTTGAG3' | 59 ℃ |

**Supplementary Table 3.** Primer used for real-time quantitative RT-PCR.

| Gene | Forward primer | Reverse primer |
| --- | --- | --- |
| *PmPP2A* | 5'AGGGTTCGGCTCGCAATAATAGA3' | 5'TGTTAGCAGCAGCATCACGAAT3' |
| *PmDAM1* | 5'AGTATGAAGGATGTTATTCAA3' | 5'CTTAAGTTCCTTGCTCAATCT3' |
| *PmDAM2* | 5'AACCAGCTACGGCAGAGGATG3' | 5'AGATTCAGATGACATGCCTT3' |
| *PmDAM3* | 5'TCGGATTGAGCAAGGAACTGG3' | 5'CATTCTCAGTTCTTCCTTTGT3' |
| *PmDAM4* | 5'ACCCTTGTCCGTGTGATGGAA3' | 5'ATCACCATCTGATTGTTGCCT3' |
| *PmDAM5* | 5'AGGCTGAATAATAATATTGAA3' | 5'TTAACGCCCCAGTTTGAGAGA3' |
| *PmDAM6* | 5'AACCAACAACCAGTTAAGGCATA3' | 5'CAATTACGGCAGATTCAGATGA3' |

**Supplementary Table 4.** Primer used for subcellular localization.

| **Gene** | **Sequence 5’-3’** |
| --- | --- |
| 1300-PmDAM1-F | AGGGGCCCGGGGTCGACATGAAAATGATGAGGGAGAAG |
| 1300-PmDAM1-R | GGTACCGGATCCACTAGTTGGAAGCCCCAGTTTGAG |
| 1300-PmDAM2-F | AGGGGCCCGGGGTCGACATGGTGAAGACGATGAGGAAG |
| 1300-PmDAM2-R | GGTACCGGATCCACTAGTGGGAAGCCCCAGTTTGAG |
| 1300-PmDAM3-F | AGGGGCCCGGGGTCGACATGATGAGGAAGAAGATCAAG |
| 1300-PmDAM3-R | GGTACCGGATCCACTAGTAGGAAGCCCCAGTTCGAG |
| 1300-PmDAM4-F | AGGGGCCCGGGGTCGACATGGTGAAAATGATGAGGGAG |
| 1300-PmDAM4-R | GGTACCGGATCCACTAGTGGAACGCCCCAGTTTGAG |
| 1300-PmDAM5-F | AGGGGCCCGGGGTCGACATGATGAATAAGATCAAGATC |
| 1300-PmDAM5-R | GGTACCGGATCCACTAGTACGCCCCAGTTTGAGAGA |
| 1300-PmDAM6-F | AGGGGCCCGGGGTCGACATGGTGAAAATGATGAGGGAG |
| 1300-PmDAM6-R | GGTACCGGATCCACTAGTGGGAAGCCCCAGTTTGAG |

**Supplementary Table 5.** Primer used in PCR reaction for Y2H assays.

| **Gene** | **Sequence 5’-3’** | **vector** |
| --- | --- | --- |
| BK-PmDAM1-F | CATGGAGGCCGAATTCATGAAAATGATGAGGGAGAAG | pGBKT7 |
| BK-PmDAM1-R | GCAGGTCGACGGATCCTTATGGAAGCCCCAGTTTGAG |  |
| BK-PmDAM2-F | CATGGAGGCCGAATTCATGGTGAAGACGATGAGGAAG | pGBKT7 |
| BK-PmDAM2-R | GCAGGTCGACGGATCCTTAGGGAAGCCCCAGTTTGAG |  |
| BK-PmDAM3-F | CATGGAGGCCGAATTCATGATGAGGAAGAAGATCAAG | pGBKT7 |
| BK-PmDAM3-R | GCAGGTCGACGGATCCTTAAGGAAGCCCCAGTTCGAG |  |
| BK-PmDAM4-F | CATGGAGGCCGAATTCATGGTGAAAATGATGAGGGAG | pGBKT7 |
| BK-PmDAM4-R | GCAGGTCGACGGATCCTTAGGAACGCCCCAGTTTGAG |  |
| BK-PmDAM5-F | CATGGAGGCCGAATTCATGATGAATAAGATCAAGATC | pGBKT7 |
| BK-PmDAM5-R | GCAGGTCGACGGATCCTTAACGCCCCAGTTTGAGAGA |  |
| BK-PmDAM6-F | CATGGAGGCCGAATTCATGGTGAAAATGATGAGGGAG | pGBKT7 |
| BK-PmDAM6-R | GCAGGTCGACGGATCCCTAGGGAAGCCCCAGTTTGAG |  |
| AD-PmDAM1-F | GGAGGCCAGTGAATTCATGAAAATGATGAGGGAGAAG | pGADT7 |
| AD-PmDAM1-R | CGAGCTCGATGGATCCTTATGGAAGCCCCAGTTTGAG |  |
| AD-PmDAM2-F | GGAGGCCAGTGAATTCATGGTGAAGACGATGAGGAAG | pGADT7 |
| AD-PmDAM2-R | CGAGCTCGATGGATCCTTAGGGAAGCCCCAGTTTGAG |  |
| AD-PmDAM3-F | GGAGGCCAGTGAATTCATGATGAGGAAGAAGATCAAG | pGADT7 |
| AD-PmDAM3-R | CGAGCTCGATGGATCCTTAAGGAAGCCCCAGTTCGAG |  |
| AD-PmDAM4-F | GGAGGCCAGTGAATTCATGGTGAAAATGATGAGGGAG | pGADT7 |
| AD-PmDAM4-R | CGAGCTCGATGGATCCTTAGGAACGCCCCAGTTTGAG |  |
| AD-PmDAM5-F | GGAGGCCAGTGAATTCATGATGAATAAGATCAAGATC | pGADT7 |
| AD-PmDAM5-R | CGAGCTCGATGGATCCTTAACGCCCCAGTTTGAGAGA |  |
| AD-PmDAM6-F | GGAGGCCAGTGAATTCATGGTGAAAATGATGAGGGAG | pGADT7 |
| AD-PmDAM6-R | CGAGCTCGATGGATCCCTAGGGAAGCCCCAGTTTGAG |  |

**Supplementary Table 6.** Primer used for BiFC assays.

| **Gene** | **Sequence 5’-3’** |
| --- | --- |
| BiFC-PmDAM1-F | TGCAGGGAGGAGGATCCATGAAAATGATGAGGGAGAAG |
| BiFC-PmDAM1-R | CGGTGCACTAGTGTCGACTGGAAGCCCCAGTTTGAG |
| BiFC-PmDAM2-F | TGCAGGGAGGAGGATCCATGGTGAAGACGATGAGGAAG |
| BiFC-PmDAM2-R | CGGTGCACTAGTGTCGACGGGAAGCCCCAGTTTGAG |
| BiFC-PmDAM3-F | TGCAGGGAGGAGGATCCATGATGAGGAAGAAGATCAAG |
| BiFC-PmDAM3-R | CGGTGCACTAGTGTCGACAGGAAGCCCCAGTTCGAG |
| BiFC-PmDAM4-F | TGCAGGGAGGAGGATCCATGGTGAAAATGATGAGGGAG |
| BiFC-PmDAM4-R | CGGTGCACTAGTGTCGACGGAACGCCCCAGTTTGAG |
| BiFC-PmDAM5-F | TGCAGGGAGGAGGATCCATGATGAATAAGATCAAGATC |
| BiFC-PmDAM5-R | CGGTGCACTAGTGTCGACACGCCCCAGTTTGAGAGA |
| BiFC-PmDAM6-F | TGCAGGGAGGAGGATCCATGGTGAAAATGATGAGGGAG |
| BiFC-PmDAM6-R | CGGTGCACTAGTGTCGACGGGAAGCCCCAGTTTGAG |

# Supplementary Data

**Supplementary Data 1.** The accession numbers of six *PmDAMs*.

PmDAM1 KY088049;

PmDAM2 KY088050;

PmDAM3 KY088051;

PmDAM4 KY088052;

PmDAM5 KY088053;

PmDAM6 KY088054.

**Supplementary Data 2.** The GeneBank accession numbers of *DAM* genes from *P. mume* and other species genes used in Multiple sequences alignment are as follows: PpDAM1 (*P. persica*, ABJ96361.2); PpDAM2 (*P. persica*, ABJ96370.1); PpDAM3 (*P. persica*, ABJ96364.1); PpDAM4 (*P. persica*, ABJ96365.1); PpDAM5 (*P. persica*, ABJ96366.1); PpDAM6 (*P. persica*, ABJ96367.1); PpsDAM3 (*P. pseudocerasus*, AIU94275.1); PpsDAM4 (*P. pseudocerasus*, AIU94276.1); PpsDAM5 (*P. pseudocerasus*, AIU94277.1); PpsDAM6 (*P. pseudocerasus*, AIU94278.1); PpyDAM1 (*P. pyrifolia*, BAM74184.1); PpyDAM2 (*P. pyrifolia*, BAM74183.1); PpyDAM3 (*P. pyrifolia*, BAM74167.1); PpyDAM4 (*P. pyrifolia*, BAM74166.1); PpyDAM5 (*P. pyrifolia*, BAI48075.1); PpyDAM6 (*P. pyrifolia*, BAI48074.1); MdDAM1 (*M. domestica*, AJW82923.1); MdDAM2 (*M. domestica*, AJW82922.1); MdDAM3 (*M. domestica*, AJW82921.1); CsDAM1 (*C. sinensis*, AIK35210.1); CsDAM2 (*C. sinensis*, AIK35209.1).

**Supplementary Data 3.** The proteins sequences of DAM genes and *P. mume* MADS-box genes.

>PmDAM1

MKMMREKIKIKKIDNLPARQVTFSKRRRGIFKKAAELSVLCESEVAVVIFSATGKLFDYSSSSMKDVIERYQAHINGGEKFNERSIELQPEYENHIRLSKELKEKSRQLRQMKGEDLEELNFDELQKLEQLVDASLGRVIETKDERIMSEIMALERKRAELVKANKQLRQRMLFRGNIGPELMKPERLNNNFGGGGEEEGMSSESATSTTCNSAPSLSLEDDSDDVTLSLKLGLP*

>PmDAM2

MVKTMRKKIKIKKIDYLPARQVTFSKRRRGIFKKAEELSVLCESEVAVVIFSATGKLFDYSSSSTKNVVERYKAHTNGVEKSDELSVELQLEIENQIRLNKELAEKSRQLRQMRGEDLEELNIDELQKLEQLVEASLGRVIETKEELIMSEIMALERKGAELVEANNQLRQRMVMLSRGNIGPGLTEPERFINNIGDGGEEGMSSESATNATISSCSSGLSLSLEDDCSDVTLALKLGLP*

>PmDAM3

MMRKKIKIKKIDCLPARQVTFSKRRRGIFKKAAELSVLCESKVAVVIFSATGKLFDYSSSSIKDVIESYKAHKNGVKKSDEPSVELQLENENHIGLSKELEEKSHQLRQMKAEDLDELNFDELQKLEQLVDTSLSRVIETKEELRMSEIMALERKGAELVEANNQLKQTMVMLSGGNTGPTLMDPERLNDNVGGGGEEEGMSSESAISTTCNSALSLSIGDDSDDVTLSLELGLP*

>PmDAM4

MMRKKIKIKKIDYLPARQVTFSKRRRGIFKKAAELSVLCESEVAVVIFSATGKLFDYSSSSIKDVIERYKARTNGVEKSDEQSLELQLENENRIKLSTELEEKNRQLRRMKGEDLEELDLDELLKLEQLVEATLVRVMETKEELIMSDIVALDKKGTELVEGNNQMVMLRDRMVMLSKRSTGPALMEPSDSATSTSCNSALSLSLEDECSDDAILSLELGRS*

>PmDAM5

MMNKIKIKKIDYLPARQVTFSKRRRGLFKKAAELSVLCESEVAVVIFSATGKLFDYSSSSTKDVIERYNADMNGVEKSNNQEIELQLENENHIKLSKELEKTSHQLRQMKGEDLEGLNLDELLKLEQLVEASLGRVMETKEELIKSEIMELERKGAELVEANSQLRQTMVMLSGGNTGPALMDPERLNNNIEGGGEEEGMSAESAISTTCNSAVSLSLEDDSSDEVTLSLKLGR*

>PmDAM6

MVKMMREKIKIKKIDYLPARQVTFSKRRRGLFKKAAELSVLCESEVAVVIFSATDKLFHYSSSSTEDVIERYKAHTGGAEKSDKQFLELQLENENNIKLSKELEEKSRQLRQMKGEDLEGLNLDELLKLEQVVEASLGRVIETKEELIMSAIMALEKKGAELVETNNQLRHRMVMLSGGNTGPAFVEPETLITNVGGGGREDDMSSESAVIATSTSCNSAFSLSLEDDCSDVTLSLKLGLP*

>PpDAM1

MKMTREKIKIKKIDNLPARQVTFSKRRRGIFKKAAELSVLCESEVAVVIFSATGKLFDYSSSSMKDVIERYQEHINGAEKFDEPSIELQPEKENHIRLSKELEEKSRQLRQMKGEDLEELNFDELQKLEQLVDASLGRVIETKDELIMSEIMALKRKRAELVEANKQLRQRASNYHNHMLSRGNIGPALMEPERLNNNIGGGGEEEGMSSESATSTTCNSAPSLSLEDDSDDVTLSLKLGLP*

>PpDAM2

MVKTMRKKIKIKKIDYLPARQVTFSKRRRGIFKKAEELSVLCESEVAVVIFSATGKLFDYSSSSTKDVVERYQAHTNGVEKSDEPSVELQLEIENHIRLTKELEEKSRQLRQIKGEDLEELNFDELQKLEQLVDASLGRVIETEEELIMSEIMALERKGAELVEANNQLRQRMVMLSRGNIGPAPTEPERFVNNIGGGGEEGMSSESATNATISSCSSGPSLSLEDDCSDVTLALKLGLP*

>PpDAM3

MVKMMRKKIKIKKIDYLPARQVTFSKRRRGIFKKAAELSVLCESKVAVVIFSATGKLFDYSSSSIKDVIERYKAHTNGVEKSDKPSVELQLENENQIGLSKELKEKSHQLRQMKAEDLEELNFDELQKLEQLVDASLGRVIETKEELRMSEIMALERKGAELVEANNQLRQTMMLSGGNTGPTLMEPERLSNNIGGGGEEEGMSSESAISTTCNSALSLSPSLGDDSDDVTLSLKLGLS*

>PpDAM4

MVKMMREKIKIKKIDYLPARQVTFSKRRRGIFKKAAELSVLCESEVAVVIFSATGKLFDYSSSSIKDVIERYEVRTNGVEKSDEQSLELQLENENHTKLSTELEEKNRQLRQMKGEDLEELDLDELLKLEQLVEATLVRVMETKEELIMSDIVALEKKGTELVEANNQMVMLRERMVMLSKRNTGPALMEPSESATSTSCNSALSLSLEDDCSDDVVLSLKLGLTVRAGR*

>PpDAM5

MMRNKIKIKKIDYLPARQVTFSKRRRGLFKKAAELSVLCESEVAVVIFSATGKLLDYSSSSTKDVIERYNADINGVEKLNNQEIELQLENENHIKLSKELEEKSRQLRQMKGEDLEGLNLDELLKLEQLVEASLGRVMETKEELIKSEIMALERKGTELVEANNQLRQTMVMLSGGNTGPALMDPERLNNNIEGGGEEEGMSAESAISTTCNSAVSLSLEDDSSDEVTLSLKLGR*

>PpDAM6

MMREKIKIKKIDYLPARQVTFSKRRRGLFKKAAELSVLCESEVAVIIFSATDKLFDYSSSSTEDVIERYKAHTNDLEKSNKQFLELQLENENHIKLSKELEEKSRQLRQMKGEDLQGLNMDELLKLEQLVEASLGRVIETKEELIMSEIMALEKKGAELVEANNQLRQKMAMLSGGNTGPAFVEPETLITNVGGGGEEDGMSSESAIIATSTSCNSAHSLSLEDDCSDVTLSLKLGLP*

>PpyDAM1

MVKRMKEKIKIRRIDYLPARQVTFSKRSRGILKKAEELSILCEAEVAVIIFSQTGKLFDYSSSSTKDVIARYKSHTGGEKWDQITLHQLQLEKENTIRLGKELEDKTRKLRQMKGEDLQDLDLDQLNKLEKLVKASIGRVIKTKEKKIMSEIMEHANKGAELIKANNQLKQRMVMLSAGGDIGPAGIMELDNLNNVGEEGVTSESATNVTTCSTSAFSLEDDCSDILSLKLGLP*

>PpyDAM2

MKIKIRKIDYLPARQVTFSKRRRGIFKKAGELSILCESEVAVIIFSQTGKLFDFSSSSTKDVIARYNSHVGGEKSDQPTLHQLQLEKENNIRLSKELEDKSCKLRQMKGVDLEDLDLDELQKLEKLVEASLGRVIQTKEEKITSDVMALEKKGAELIEANNQLSQKMVMLPGGDSGPEAILNNIGEESVTSESATNVTTFSNSSLSLEDDCSDTLSLKLGLP*

>PpyDAM3

MVKRMNEKIKIRRIDYLPARQVTFSKRRRGIFKKAEELSILCESEVAVIIFSQTGKLFDYSSSSTKDVIARYKLHTGGEKSDQITLHQLQSEKENTIRLSKELEDKTRKLRQMKGEDLQDLDLYQLNKLEKLVEASVGRVIKTKEKKIMSEIMALTNKGAELIEANNQLKQRLVMLSARGDIEPAAIMELENLNNVGEEGMTSESATNVTACSSSALSLEDDCSDILSLKLGLP*

>PpyDAM4

MVERMKEKIKIRRIDYLPARQVTFSKRSRGILKKAEELSILCEAEVAVIIFSQTGKLFDYSSSSTKDVIARYKSHTGGEKWDQITLHQLQLEKENTMRLSKELEDKTRKLRQMKGEDLQDLDLDQLNKLEKLVEASIGRVIKTKKKKIMSEIMAHANKGAELIDANNQLKQRVVMLSAGGDIGPAGIMELDNLNNVGEEGVTSESATNVTTCSSSAFSLEDDCSDILSLKLGLP*

>PpyDAM5

MVKRMNEKIKIRRIDYLPARQVTFSKRRRGIFKKAEELSILCESEVAVIIFSQTGKLFDYSSSSTKDVIARYKLHTGGEKSDQITLHQLQSEKENTIRLSKELEDKTRKLRQMKGEDLQDLDLYQLNKLEKLVEASVGRVIKTKEKKIMSEIMALTNKGAELIEANNQLKQRLVMLSARGDIEPAAIMELENLNNVGEEGMTSESATNVTACSSSALSLEDDCSDILSLKLGLP*

>PpyDAM6

MKIKIRKIDYLPARQVTFSKRRRGIFKKAGELSILCESEVAVIIFSQTGKLFDFSSSSTKDVIARYNSHVGGEKSDQPTLHQLLLEKENNIRLSKELEDKSCKLRQMKGVDLEDLDLGELQKLEKLVEASLGRVIQTKEEKITSEVMALEKKGAELIEANNQLSQKMVMLPGGDSGPEAILELENLNNIGEGSVTSESATNVTTFSNSSLSLEDDCSDTLSLKLGLP*

>PpsDAM3

MVKMMRKKIKIKKIDSLPARQVTFSKRRRGIFKKAAELSVLCESKVAVVIFSATGKLFDYSSSSTKDVIERYKAHTNGVEKSDKPSVELQLENENHIGLSNELEEKSHQLRQMKAEDLEELNFDELQQLEQLVDASLGRVIETKEELRMSEIMALERKGAELVEANNQLRQTVSNHHNHMVMLSGGNTGPELMEPERLNNNIGGGGEEEGMSTESAISTTCNSAHSLGDDSGNVILSLKLGLP*

>PpsDAM4

MVKMKREKIKIKKIDYLPARQVTFSKRRRGIFKKAAELSVLCESEVAVVIFSPTGKLFDYSSSSVKDVIKRYKARANGVEKSEESLELQLEHENRIKLSKELEEKNSQLRKMKGEDLEELDLDELLKLEKLVEATLVRVMETKEELIMSDIMVLEKKGTALVEANNQMVMLKERMVMLSKRNTGPELMEPSESATSTSCNSALSLSLEDDCSDDVILSLKLGRP*

>PpsDAM5

MVRNKIKIKKIDYLPARQVTFSKRRRGLFKKAAELSVLCESEVAVVIFSATGKLFDYSSSSTKDVIERYNVHMNDVDKLNDQEIELQLENENHIKLSKELEEKSRQLRQMKGDDLEGLNLDELLKLEQLVEASLGRVMETKEELIKSEIMELERKGAELVEANNQLRQTMVMLTGGNTGPALMDPERLNNNIGGGGEEEGMSAESAISTTCNSAVSLSLEDDSSDEVTLSLKLGR*

>PpsDAM6

MVKMMREKIKIKKIDYLPARQVTFSKRRRGLFKKAAELSVLCESEVAVVIFSATGKLFDYSSSSIEDVIERYKAHTNGVQKSNKQFLELQLENENHIKLSKELEEKSRQLRQMKGEDLEGLNLDELLKLEQLVEGSLGRVIETKEELIMSEIIALEKKGAELVETNNQLRQRMAMLSGGNTGPALVEPETLNTNIGGGGEDGMSSESATMATSTSCNSALSLSLEDDCSDVTLSLKLGLP*

>MdDAM1

MAREKIQIKKIDNATARQVTFSKRRRGLFKKAEELSVLCDADIALIIFSSTGKLFEYASSSMKEILERHNLHSKNLDKLEQPSLELQLVENSNYSRLSKEIAAKSHQLRQMRGEEIQGLSLEELQQLEKSLEAGLGGVVEKKSEKIMKEINDLQRNMNVQAMQLTEENERLRQQVVEKSNGRRLVHVDSENLITEEGQSSESVTNLCKSNSGPQDYDNSVTSLKLGCA*

>MdDAM2

MVKIRKEKIKIRRIDYLPARQVTFSKRRRGIFKKAEELSILCESEVAVIIFSQTGKLFDFSSSSWKRDIIHVNLIKLWSSPPDNQGELTSFPMFGIGQGHRNTKDVIARYKSHTGEKSNQSMLDQLQLEKENTIRLSKELEDKTRKLRHLKGEELQDLDLDELQKLEKLVEASHGRVMETKGDELVEANNQLKQRMVMLSARGDIGPAAIMELENLNNGGEEGVTSESATNVTTSSNSPLSLEDDCSDILSLKLGCAALTS

>MdDAM3

MKIKIKKIDYLPARQVTFSKRRRGIFKKAGELSILCESEVAVIIFSQTGKLFDFSSSRLWSSPPXNQDELVSFPFVDSHAIESDKLIMHVNRRTKDVIARYNSHIGGEKSDQPTIHQLQLEKENNIRLRKELEDKSCKLRQMKGVDLEDLDLDELQKLEKLVEASLGRVIQTKGAELIEANNQLSHRMVMYPRGDIGPEAILELENLNNIGEESXTSESTTNVTTCSNSSLSLEDDCSDILSLKLG

>CsDAM1

MVRQRIQIKKIDNVTSRQVTFSKRRKGLFKKAQELSTLCDAEIALIVFSATGKLFEFSSSSMRQVIERHNLESGNLVNLNQPSLEQQLENSGCTILSKEVNKKIHELRQLRGEELQGLDAEELKNLEKSLEGGLSRVLKTKGEIMEKEITARERKEARLVEENVWLKQKVPMEIVKIGQTHDDQQGQSAEFITNNGSSAAPPQDNDSSDTSLKLGLPFPELN

>CsDAM2

MVRQRIQIKKIDNVTARQVTFSKRRRGLFKKAHELSTLCDAEIALIVFSATGRLFEYASSSTRQVIERHNLQPQNLVQLNQPSLELQLENSTRAMLSKEAEERTLELRQLRGEELHELGFEELKKLEKSLEGGLSRVLKTKDDRVEKEIAALRRKEARLMEENAWSKQQVQMQIVNMGQPQEQGQSSESITNNGSTVAPPQDYDSSDTSLKLGLPYQS

>PmMADS01

MAREKIQIKKIDNATARQVTFSKRRRGLFKKAQELSVLCDADIALIIFSSTGKLFEYASSSTKEILERHNLHAKNLSKIEQPSLELQLVENSNYSALSKEITAQSQQLRQIRGEDIQGLNLEELQQLEKSLEAGLGRVIEKKGEKIMKEISDLESNAMRLVEENERLRQQVLEKHNSQRPVRADSENMVMEEGQSSESVTTNLCNSNSAPQDYESSDTSLKLGLPYSG*

>PmMADS03

MSEGSIEFILLNVGLQIKMGRGKIEIKRIENTTNRQVTFCKRRNGLLKKAYELSILCDAEVALIVFSSRGRLYEYSNNNIRNTIERYKKACSDSSGSTSITEINAQYYQQESAKLRQQIQMLQNSNRHLMGDALSTLSVKELKQLENRLERGINRIRSKKHEMLLAEIEYLQKKEIELENENVCLRTKISEVERLQQANMVGPELNAIQALASRNFFSQTMMEGGATYPQQDKKILHLG*

>PmMADS15

MAYENKSMSLDSPQRKLGRGKIEIKRIENTTNRQVTFCKRRNGLLKKAYELSVLCDAEVALIVFSNRGRLYEYANNSVKETIERYKKACAESTNTGSVSEASTQYYQQEAAKLRAQIGNLQNSSRHMMGESLSSMNMKDLKNLESKLEKGINRIRSKKNELLFAEIEYMQKREIDLHNNNQLLRAKIAENERSQQNINVMAGGGSYEIMQSQPYDSRNYFQVNALQPNHQYNSRQDPMALQLV*

>PmMADS12

MGRGKIEIKRIENSSNRQVTYSKRRNGIIKKAKEITVLCDAKVSLVIFASSGKMVEYCSPSVTVTDILDKYHGQAGKKLWDAKHENLSNEVDRVKKDNDSMQVELRHLKGEDITSLTHKELMALEDALENGLASIRDKKSKFVDILRENERALEEEHKRLTYELHKQEMKIEENVRELENGYHQRLGNYNNQIPFAFRVQPIQPNLQERM*

>PmMADS13

MTRGKIQIKRIENATNRQVTYSKRRNGLFKKAHELTVLCDATVSLIMVSSSGKIHEYISPSTTTKQFFDQFQKTKGVDIWSSHYEVSHSSLLSLSLSLSLSLSIYIYMYIIVWLCVQAMQEHLKKLKEVNRSLQKQIRQRVLGECLNDMSFDELRGVEQEMEGAVDVIRKRKVDARDDTHYRLVENGGEDYESAFGYSSNGGPRIFA*

>PmMADS14

MGRGRVELKRIENKINRQVTFAKRRNGLLKKAYELSVLCEAEVALIIFSNRGKLYEFCSSSSMLKTLERYQKCNYGAPETNVSAREALELSSQQEYLKLKARYEALQRNQRNLLGEDLGPLSSKELESLERQLDMSLKQIRSTRTQCMLDQLTDLQRKEHMLNEANKTLKQRLFEGYHVNSLQMNPNADEYGRQQAQAHGDGFFHPLDCEPTLQIGYQNDPISVVTAGPSVSNYMAGWLP*

>PmMADS17

MGRGRVELKRIENKINRQVTFAKRRNGLLKKAYELSILCDAEVALIIFSNRGKLYEFCSSSSSILKTLERYQKCSYGQVEVNKPAKELEQSSYREYLKLKGRFESLQRTQRNLLGEELGPLNTKELEQLERQLESSLKQVRSTKTQYMLDQLSDLQNKEQMLIEANRDLSLKLDDISSRNQIRQSWEGGNQGGMAYGTQHAQSQGFFQPLDCNPTLQIGYSNVGSEQMSATTHAQQVNGFIPGWML*

>PmMADS18

MGRGKVQLKRIENKISRQVTFSKRRAGLLKKAHEISVLCDADVALIVFSTKGKLFEYSSDSSMESILERYDQYSHAEQQLTTDFDPQGSCWSLEYPKLAARIEVLQRKLRHFTGEDLDSLSLRELQNLELQLETALKRIRTRKNQLMHESISVLHKKQKALQEQNNSLGKKLKEKENMLEVEHDGQVQQVEQHQSNQAAHNSSTLMLMPPPPPQTSSTPALLASLTIGGGIQARGGMEDGDDNDGRTQTRPPPTTNTLMPMWMYRHFNE*

>PmMADS20

MGRGRVELKRIENKINRQVTFSKRRNGLLKKAYELSVLCDAEVALIIFSSRGKLHEFGSAGFIWILSLPIKTSNEAVASQVLEAPEMKSCSSYLLSSRSMQSWYQEVTKLKAKYESLLRTQRQLLGEDLGPLNVKELQNLEKQLEGALAQARQRKTQLMIEQMEDLRKKERHLGDLNKQLRVKLETEGQNLKAIQNMWSSNAAAGSSSFSFHSSQTNPMDCQPHEPVLQIGYHQYLPVEGPSMSKSMACETNFIQGWVL*

>PmMADS21

MVRGKTQMRRIENATSRQVTFSKRRSGLLKKAFELSVLCDAEVALIIFSPRGKLYEFASSSMQTTIERYQKHTKDNLTNNKSVSTDQNMQHLKQESSSMMKQIELLEVSKRKLLGEGLGSCSIEELQEIEQQLERSVSNVRARKTQVFKEQIEQLREKGKALAAENEKLIEKCGRIQPRQASNEQRENLAYTESSPSSDVETELFIGLPERRKR*

>PmMADS22

MVRGKIEMKRIENATSRQVTFSKRRNGLLKKAFELSVLCDAQVSVIIFSQKGRLYEFSSSDMQETIKRYHKHAKAGQTNKIEVEEYVEQLKHESTAMAKKIENLEASQRKLLGHGLDSCSVEELQEITGQLERSVRKIRERKAHLFAEQMEQLRAKERLLLEENAKLSEEFGAQPRLLLQQQQLSVEEKGAVSYWSLSSPSSEVETELFIGPPVTRC*

>PmMADS23

MGRRKVVLERIENKINRQVTFSKRRNGLLKKAYELSVLCDAQVALIISSSRGKLYEFGSTDYNSVHSLISVNKILERYRQCCYSLQGNVAENETQNLYQEVSKLKVKYESLQLSQRHLLGEDLEKLRLKELVNLENQLDKTLSKARQRKVLVICVM*

>PmMADS24

MTRRKIQIKKIDNTTARQVTFSKRRRGLFKKAQELSTLCDAEIALVVFSATGKLFEFTSSSVQQVIERHHLLSSDFDKLNHPSLELQSFCMSQLESSTSAALSKEIAEKTHELRKLRGEELQELNMKELQELEKLLGSGLRRVRDAKCEIVLKEITSLKWKGSQLMQENKRLKQMENRQVQTLELEQGQSSEPIGDFIHSDPSQDHDSSDISLKLGLAFPSGI*

>PmMADS25

MVRGKTQMKRIENAASRQVTFSKRRNGLLKKAFELSVLCDAEVALIIFSTRGKLYEFSSSRRFSIGNTLDRYQKRVKDQGLGSKAVQVDMEHGKDDTSSMAKKIDFIEASKQKLLGNCLESCSIEELQQTENQLERSLSKIRARKTQLLREQIENLKEEEKNLFEQNAKLREKCGMQPLGPPSARKDEENCAVRQPRTPDMEDVETDLVIGPPERRRSSQNP*

>PmMADS27

MGRGRVQLKRIENKINRQVTFSKRRSGLLKKAQEISVLCDAEVALIVFSTKGKLFEYSTDSCMERILERYERYSYSEKQLLANDNESTGSWTLEHAKLKARVEVLQRNHSHFMGEDLQSLSLKELQNLEQQLDSALKHIRSRKNQVMYESISELQKKDKALQEQNNLLAKKVKEKEKALAPQAQSWEQQVQNQGLDCSSTLLPEALHSGSNYQGIRSDGFGGDHEDENETPTANRPNTLLPPWMLRHLNE*

>PmMADS28

MGRGRVELKRIENKINRQVTFAKRRNGLLKKAYELSVLCDAEVALIIFSSRGKLYEFCSSMSMLKTLEKYQRCSYGSLEANRPVNDTQKQNSYQEYLKLKARVEVLQQSQRNLLGEDLAPLNTKELEQLEHQLEASLNQIRSTKTQFMLDQLCDLQNKEQMLVEANKALRRKLEETSGQAPPPLAWEAAGHGNNNDQHTRLPHHPHSQGFFHPLGNNSTSQIGYTPLGSDHHEQMNVGNHGQHVNGFIPGWML*

>PmMADS29

MGRGKIEIKLIENHTNRQVTYSKRRNGIFKKAQELTVLCDAKVSLIMLSNTGKMHETKRMYDDYQKTLGVDLWSSHYQAMKDTLWKLKEINNKLRREIRQRLGHDLNGLTYEQLHSLEDKMASSLEAIRERKYHVLKTQMETYKKKVKNLQERRGNMLHGYFDQEVASEDPQYGYVDNEGDYESAVALANGASNLFTIHLHQDIRDHANLHHHGGSSLGSSITHLHDLRLA*

>PmMADS31

MGRGRVQLKRIENKINRQVTFSKRRTGLLKKAHEISVLCDAQVALVVFSNKGKLFEYATDSCMDQILDRYERYSYAERQLVEPDFESQCNWTFEYSRLKAKVELLQRNQRHYLGEDLDSLTLKEIQSLEHQLETALKQIRLRKNQLMHESISELQRKERAMQEQNNLLAKKIKEKEKAAAEEVHNWEQQNNGLNMLPQPLPCLNMGGTQQDEFLQVRRNQLDLTLEPLYSCNLGCFAA*

>PmMADS32

MGRGKVELKRIENKINQQVTFAKRRNGLLKKAYELSVLCDAEVALIVFSTRGKLYEFCSGSSMEKTLERYQRCSYSALEASQPAQDSQSRYQDYVNLKAKVEVLQLTQRNFLGEDLGHLGTKELQQLENQLDMSLRQIRSTKTQVMHGQISDLLRKEQMLLEANNELRRKLEECDAAIERYSWTTEEQNQNVPNSSHHQAAQFEGVLDHSQCNNTLQIGYNPAAVTDHHELQSSTQSHSGLIFPGTWVL*
